# The combined positive impact of Lean methodology and Ventana Symphony autostainer on histology lab workflow

**DOI:** 10.1186/1472-6890-10-2

**Published:** 2010-02-08

**Authors:** Lewis A Hassell, Crystal F Glass, Clinton Yip, Patricia A Eneff

**Affiliations:** 1 University of Oklahoma Health Sciences Center, Oklahoma City, Oklahoma, USA; 2 OU Medical Center, Oklahoma City, Oklahoma, USA; 3 Ventana Medical Systems, Tucson, AZ, USA

## Abstract

**Background:**

Histologic samples all funnel through the H&E microtomy staining area. Here manual processes intersect with semi-automated processes creating a bottleneck. We compare alternate work processes in anatomic pathology primarily in the H&E staining work cell.

**Methods:**

We established a baseline measure of H&E process impact on personnel, information management and sample flow from historical workload and production data and direct observation. We compared this to performance after implementing initial Lean process modifications, including workstation reorganization, equipment relocation and workflow levelling, and the Ventana Symphony stainer to assess the impact on productivity in the H&E staining work cell.

**Results:**

Average time from gross station to assembled case decreased by 2.9 hours (12%). Total process turnaround time (TAT) exclusive of processor schedule changes decreased 48 minutes/case (4%). Mean quarterly productivity increased 8.5% with the new methods. Process redesign reduced the number of manual steps from 219 to 182, a 17% reduction. Specimen travel distance was reduced from 773 ft/case to 395 ft/case (49%) overall, and from 92 to 53 ft/case in the H&E cell (42% improvement).

**Conclusions:**

Implementation of Lean methods in the H&E work cell of histology can result in improved productivity, improved through-put and case availability parameters including TAT.

## Background

The core process of every anatomic pathology laboratory has been the hematoxylin and eosin (H&E) stain for well over 100 years. Sequential processing steps involved in the diagnostic preparation of tissue--gross dissection, selection of tissue blocks, processing, imbedding in paraffin, cutting and mounting on glass slides, all funnel through the final stages of staining, and cover-slipping in preparation for review and diagnosis by a pathologist. Efforts at automating or otherwise improving portions of these processes over the past century have yielded some significant gains, but have also at times introduced potential for variation, defects, and delays. In not every case have these been adequately studied before and after changes have been introduced. A combination of economic pressures to increase productivity in order to maintain profitability, the need to hasten the time to diagnosis while preserving ample time for study and evaluation by pathologists and trainees, and the desire to reduce sources of medical error have entailed upon the histology laboratory the classic "rock and hard place" logjam that cannot be solved by reliance upon the status quo methods of the past. Industrial productivity and quality systems methods such as Lean and Six Sigma have been introduced into healthcare and laboratory operations over the past several years to successfully confront this dilemma [1-4].

One of the problems in comparing practices between top-performing labs, even among closely defined areas of a lab such as histology, is that the metrics of measurement are not sufficiently standardized to compensate for all the variables impacting productivity and are subject to considerable variation according to case mix and other local circumstances. However, in undertaking process changes, internal benchmarks based on past performances can be utilized effectively as an evaluation measure [5]. Whether such results can be matched elsewhere may depend on a variety of factors including those we have been able to identify, and others we have not.

Lean methodology, adopted from industrial production experience, has recently been advocated as a means to improve laboratory financial performance while also generating progress in patient safety. In reducing the several types of waste inherent in most systems, the reduced number of process steps and better-designed "goof-proof" processes reduce variability and potential for errors that may result in harm to patients. Several publications documenting application of these tools to laboratory processes, including histology processes, have shown significant results [6,7]. While often quite straight-forward and low-tech, Lean concepts are also being embraced by vendors offering histology equipment and solutions, which create new potential synergies in performance.

In this paper, we present data from our experience in combining low-tech Lean process improvements, workspace redesign, and a Lean-engineered automated staining system, to the H&E workflow cell.

## Methods

The histology laboratory at OU Medical Center operates 24 hours a day, six days a week, supporting the histology needs for the OU Medical Center, The Children's Hospital at OU Medical Center, the OU Physicians, Oklahoma State Health Department, OU Oral Pathology and some on-campus research operations. The need to improve TAT for the benefit of patient care and fiscal performance, together with our efforts to maintain or improve the time trainees have to review case material prior to sign-out, impelled us to look for tools like Lean to help us meet the demands of our practice situation. Also, volume growth (aka market share) is an important goal of the medical center, which has worked to improve through-put in the operating room suites, and has plans for additional out-patient procedure areas. While accession volume over the recent past has seen only limited growth, on-campus expansion is occurring as a new cancer center and an ambulatory surgery center are nearing completion and additional physician space created. It is expected that the slope of the growth curve will shift upwards. Space constraints, in addition to the ubiquitous budgetary pressures, limit the histology lab's ability to add personnel beyond the seven full time equivalent (FTE) employees currently devoted to routine H&E production.

We used non-concurrent production data to assess productivity under initial and redesigned systems of workflow. Raw block and slide count data as tabulated monthly were combined with worked hours and normalized to produce a productivity ratio to track overall process production (Number of blocks + number of slides/[total hours worked/173 hours/FTE]). Only employees engaged in the core histology processes (i.e. excluding special staining procedures) were included. Other studies have also used this generic method of workload and productivity measurement for both internal and external comparison [5]. We employ this method for before and after measurements for comparison with other published studies, recognizing that our case and specimen mix is different from many other laboratories and that gross block and slide count is an imperfect measure of workload. Statistical comparison of before and after productivity was performed using the student's two tail t-test, as our measurements of this parameter show a normal (Gaussian) distribution of values over time (mean, median and mode not significantly different.) The employees engaged in this section of the laboratory did not change over the study period and the differences in volumes were negligible.

Histology section processes, through-put and capacity at baseline were directly measured by observation of sample cases on a typical day by an experienced, Lean-trained observer not employed within the laboratory, as part of an overall value stream analysis. Randomly selected cases were time stamped at each stage of progress through histology processing. Repeat time stamp random case study following process modifications were used for comparison. Within process total turnaround times were compared for the time stamped sample cases before and after process changes. Waiting times between steps were included in these measurements. Percent improvements were calculated. Cycle time (total time required to produce a single unit) and takt time (the ratio of the time available to do the work to the number of units produced or demanded in that time) measures were made per standard methods. We used daily case and slide log sheets to determine the time of delivery of the last case each day, as well as the number of cases available at earlier points in the process. The number of days per month when new cases were distributed to pathologists after 10 am was used as a measure of the robustness of the process changes to handle variations in volume and workplace or staffing variability.

Sequential step mapping with decision points and alternative pathways displayed were used to visualize opportunities for simplification or improvement. The process improvement team collaborated with vendors to identify how instrumentation changes could impact the overall flow of work. In the H&E workcell, we selected the Ventana Symphony system. This instrument integrates the baking, staining and coverslipping functions into a quality-controlled, continuously monitored and individually adjustable process that allows continuous sequential flow of trays of up to 20 slides.

**Figure 1:**
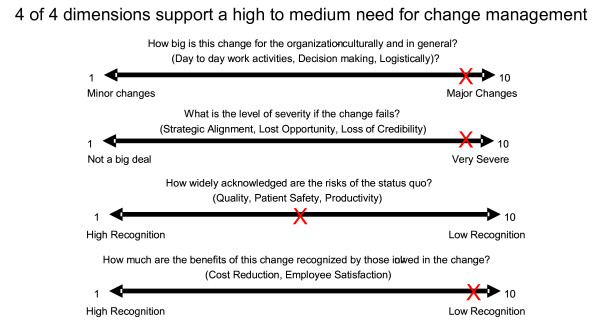
**Self-Assessment of our status on four dimensions of "change readiness"**.

Engaging histology laboratory workers in the change process is important. Measuring ourselves on the four axes of "change readiness" supported our sense that active management of the changes would be imperative to our success [8] (Figure 1). Sustained change cannot be accomplished without the involvement and participation of workers at all levels of an operation. To do this in our laboratory, we sought first the support and involvement of senior leadership, both on the physician and hospital sides of our operation. We provided data on the patient safety risks present in our current methods, education in current business and fiscal climate, and reassurances that our goal in improving efficiency was not to eliminate positions. We charged small teams across various boundaries with study and implementation of portions of the transformation. We encouraged employee input on process changes and improvements. Sometimes this meant that we encountered push-back from those who felt changes were not needed or that proposed changes would not be demonstrably better. We provided quantitative data and graphics of our current state, and where the projected changes could take us to attempt to allay active resistance and engage support.

We conducted initial and interim cost analysis of the project to determine the economic impact on operations. We used real salary and benefits data for the employees in the H&E work cell. Actual incurred costs for instrumentation changes were included, as well as overall costs for space renovation, although some of these were part of the larger workflow redesign. Cost savings or increases associated with the project were derived from actual reagent and supply costs, observed production changes and the inferred deferral of additional staff to accommodate projected growth, as well as the deferred need for space expansion or additional instrumentation to meet volume demands. In addition, cost avoidance associated with a reduction in liability was calculated from the experience of other laboratories with underwriter-reduction in liability rates following adoption of similar processes (Judy Frost, MPL, personal communication.)

## Results

**Figure 2:**
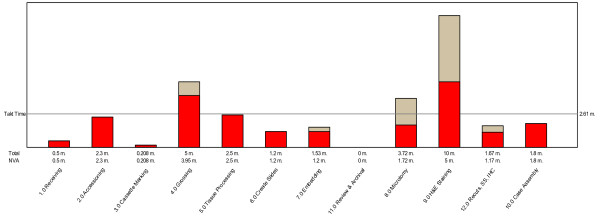
**Baseline process performance and threshold tatk time needed to match demand**.

Our baseline process studies, based on an average demand of 500 slides per day (62 cases,) revealed a takt time of 2.61 minutes. However, measurement of the capability of each process step demonstrated an inability of the status quo methods to meet this demand in the areas of gross dissection, microtomy and H&E staining. (Figure 2) As is evident from the graph, the biggest bottleneck was in the H&E staining step. While reducing non-value adding steps could bring the other processes below the takt time threshold, this alone would not work in the H&E production workcell. This workcell used a semi-automated batch stainer, an automated coverslipping device, in addition to traditional embedding stations with integrated paraffin dispenser and cold plate, and manual microtomes. Different case types were embedded in batches as they came from the tissue processor, then moved in batches to the microtomy stations where batches of slides were prepared, and the sections made. Baking and staining then ensued, also in batches. Case assembly happened as groups of finished slides came off the coverslipping device and were matched with blocks and paperwork for assembly and delivery to the pathologist.

**Figure 3:**
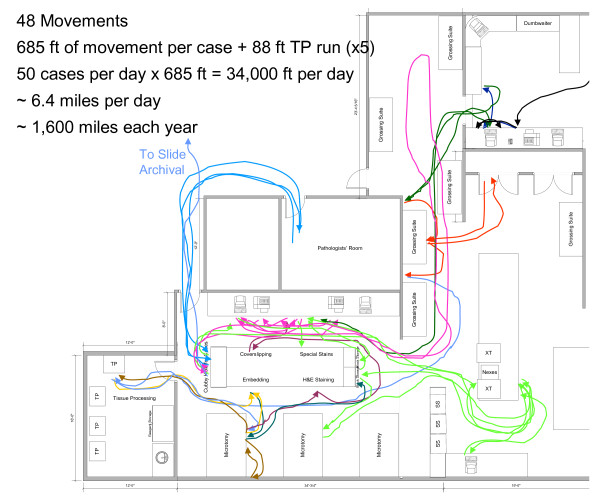
**Baseline floorplans and process flow diagram**.

**Figure 4:**
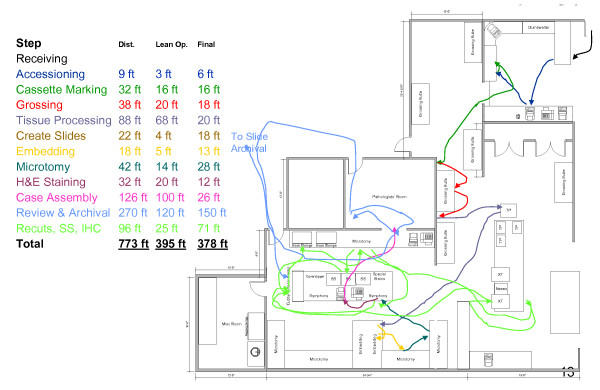
**Floorplan and process diagram following change**.

**Figure 5:**
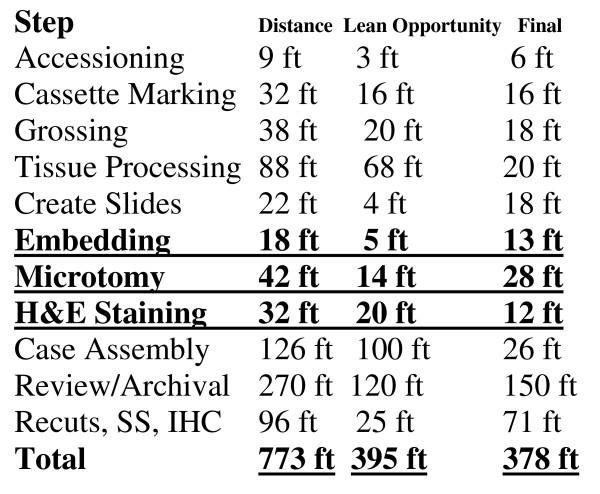
**Summary of movement savings following implementation**.

Following an implementation of the floorplan and process changes described here (Figures 3 and 4), and installation of the Symphony integrated staining/coverslipping machines, including elimination of multiple process steps that were considered non-value-adding, the cycle time for this workcell was projected to be reduced such that the overall takt time could be sustained. Total process and floorplan redesign projects substantial movement savings as detailed in Figure 5, with a breakdown of potential savings by process step.

**Figure 6:**
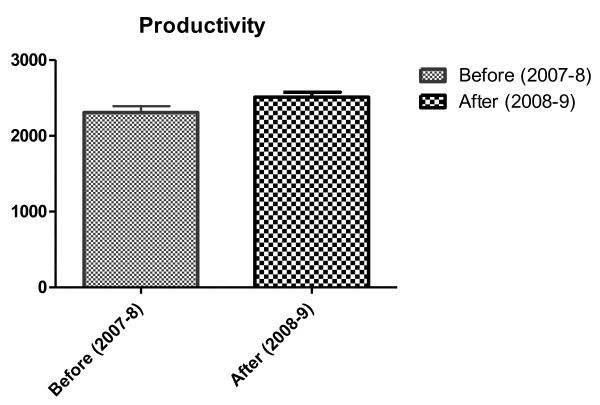
**Productivity gains from Lean implementation in H&E workcell**.

Raw measurements of productivity showed improvement in productivity data immediately following implementation of the H&E workcell modification. These results show statistical significance (Table 1 and Figure 6).

**Table 1:** Comparison of monthly productivity following H&E workcell process change

Month	Pre-change productivity (2007-8)	Post-change productivity (2008-9)
November	2515	2593
December	2374	2598
January	2041	2259
February	2404	2575
March	2209	2534
Mean (SD)	2309 (185)*	2512 (143)*

* t < 0.0005 (two-tail test)

The distance-travelled graphics in the baseline state and in the redesigned state are presented in Figure 5. Specimen movement distances were decreased within the H&E workcell from 92 to 53 feet, a reduction of 42%. These reconfigurations of workcell instrumentation and organization represented a collaborative effort of consultants, lab team members and leadership.

Time to first available slides decreased from a minimum of 12 hours to four hours. The primary intent of this was to provide added time for resident review of slides prior to sign-out, and was accomplished by use of a short sequence processor run for biopsy specimens arriving in the laboratory in the morning and a second large section run for tissues fixed overnight before cutting the following morning. By introducing these modifications 17% of the total workflow was shifted from a night-shift time to an afternoon time, and this contributed greatly to the overall change in average time from gross station to finished slides of 2.9 hours. This change however, also meant that the average time at which the last case was presented for diagnosis also improved from 9:59 to 8:55, an improvement of one hour and four minutes. The number of days in which new slides/cases were arriving on the pathologist's desk for diagnosis after 10 am was reduced 50%.

The process evaluation identified several significant wait times between steps, in addition to the bottlenecks noted above in actual process capability, some of which were eliminated or reduced by the changes implemented to date. (Table 2) These were noted to be significant barriers, and an opportunity for large gains in TAT, even if we were only able to reduce them by half. This portion of the project, focused on the last two of these pauses, was successful in reducing the measured times by 11%. Through altering the work flow and manner of work, and adding additional processor runs, the waiting times at gross dissection, case assembly and the microtome were decreased. We encountered resistance to the changed work methods calling for single piece flow embedding and cutting steps, as evidenced by persistence of a 2.5 hour wait observed between embedding and microtomy. Future anticipated process accountability changes are expected to overcome this. By using the Symphony stainer to allow continuous case flow, generally assigning a single case to a single tray, we further facilitated the process.

**Table 2:** Inventory or wait times for specimens between process steps

Process step	Wait pre-Lean	Wait post-Lean	Percent improvement
Accessioning to grossing	2.5 hours	2.5 hours	0.0%
Gross description to processor	3.5	3.0	14%
Embedding to microtomy	2.5	2.5	0.0%
Microtomy to H&E staining	1.0	0.75	25%
H&E Staining to Case Assembly	1.5	1.0	33%

**Figure 7:**
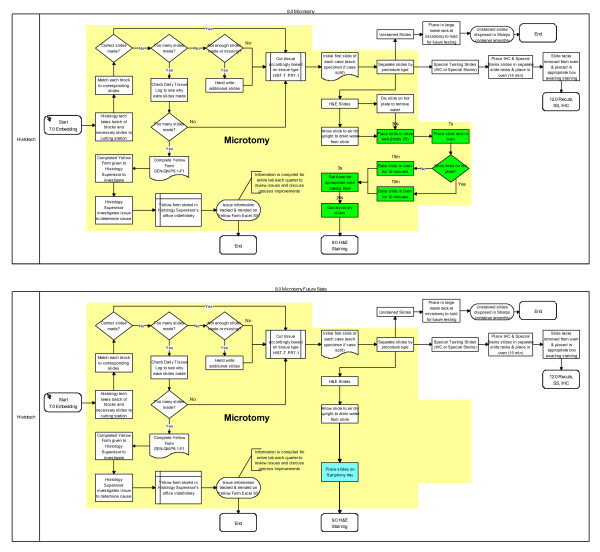
**Pre- and Post-Symphony Impacted Process Steps (in green) at the Microtomy Workbench**.

**Figure 8:**
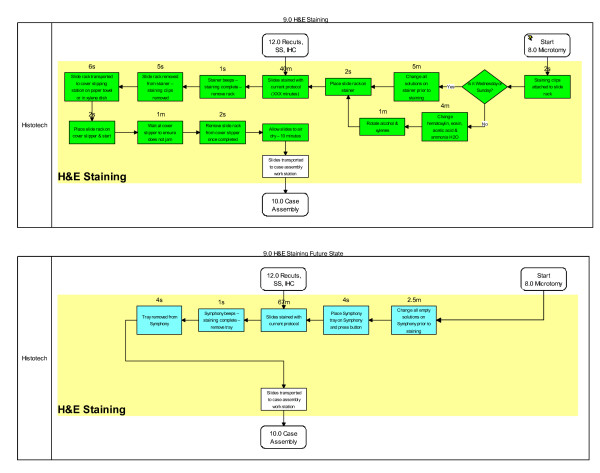
**Pre- and Post-Symphony Impacted Process Steps (in green) at the H&E Staining Workbench**.

Process simplification and worker efficiency gains from installation of the Symphony automated platform appear significant. Both the Microtomy and H&E Staining workbenches were positively impacted as illustrated in green (Figures 7 and 8). At the microtomy bench, a total of seven manual and highly variable steps are replaced by a simple single step leading to loading Symphony (Figure 7). At the H&E Staining bench, thirteen steps are now automated. A total of 23 minutes of manual and semi-automated work are absorbed by Symphony (Figures 7 and 8). Coupled with eliminating a queue area for slides that needed to go to the oven prior to staining and to the coverslipper after staining, a total of 30 minutes was saved as a result of converting the H&E staining process from a semi-automated platform to a fully automated platform. We did not capture the amount of time spent draining and changing stains in the baseline state, though we retrospectively estimate this to be just over 1 hour per week with the slide volume in our lab. With a direct waste line in the Symphony however, no manual draining of waste is needed. Additionally, over 32 feet (47%) of travel distance was removed from the Microtomy and H&E Staining processes (Figure 4) - a total of 106 miles of transport distance saved per year.

Case assembly at the tail end of the sequence had previously been a task for only the most meticulous of technologists, as the chaos introduced between grossing, processing trays, embedding stations, cutting areas, and batch strainers often meant that a case was scattered over several runs. Errors in assembly were common, despite meticulous care. Delays due to the need to hunt down an errant slide in another run were the norm. Although not measured in the baseline state, the post-change anecdotal impression is that an increased number come off the Symphony stainer complete and ready to be presented to the pathologist. Cases with delayed blocks (some case types, e.g. gynaecologic tumor debulking and staging procedures, often had smaller component specimens along with large specimens that were processed on separate processor runs from the major resection) were however still easier to reassemble because secondary portions arrive together at the end of the staining process.

The results of our cost analysis show an actual increase in our fully loaded operating cost per slide of 3.2% over baseline at our interim state, inclusive of capital costs, service contracts, renovation and reagents as well as labor. We project, based on historical volume growth rates of 2.8%, to defer the need for additional staff in the H&E workcell at least 11.6 years due to the improved productivity observed from the implemented changes to date. Another way of stating that would be that the H&E work cell has the capacity for over 32% more work before exceeding the takt threshold for any of the process steps in this work cell. We also project to defer the need for space expansion and additional instrumentation, based similarly on historic growth rates exclusive of known projects or added contracts/clients, for at least 10 years. Inclusive of these avoided costs (in 2009 dollars) and a reduction in liability costs, our cost/slide is reduced by 8.8%. The payback period from operational savings for just the improvements included in this portion of our project then becomes 3.6 years.

## Discussion

Lean production methods, originating in industrial settings such as Ford, Toyota and elsewhere, have been applied more broadly in the healthcare sector over the past decade with encouraging results. Laboratories in particular have been early adopters. Most of the reports of such endeavours to date have emphasized the gains to be had from innovative, but relatively low-tech changes to established processes. However, an important aspect of the Lean transformation of any industry is the integration all along the value chain of suppliers, equipment manufacturers and customers. (For example, a parts manufacturer that only does batches of 100,000 isn't going to mesh well with a customer who uses only 100 parts/week and will modify the product (and thus the part design) after 12 months.) This report details our experience working with one such vendor, Ventana Medical Systems, in integrating a Lean-based advanced stainer and coverslipping system, the Symphony, with other more simple Lean process modifications. Increasingly, equipment and reagent suppliers who recognize this transformation in pathology and can "right-size" products, offer "just-in-time" resupply and other Lean-integration tools will gain advantage over more traditional vendors.

Many of the gains we have achieved to date are attributable to traditional, low-tech Lean process changes such as processor scheduling, and work cell rearrangements. But despite the perceived high price on linking these with a Lean-engineered system like the Symphony stainer, we have been able to demonstrate a reasonable payback period for the capital expenditure and marginal increase in direct operating costs. We believe this illustrates the feasibility and value in integrating technical and process changes simultaneously and with deliberate forethought.

Reports of potential impacts on productivity and turnaround time have been published for some of the new tissue processors [9], but no reports to date have focused on the impact of single-piece flow H&E strainers. The quality of productivity data in histology lags behind that available for other laboratory sections, and further dialogue and development of readily interchangeable methods are needed [10,11]. Our experience shows that when combined with other Lean-oriented redesign factors, an automated, single-piece flow stainer and integrated coverslipping machine, even when measured by rather gross traditional measures, can help to increase overall productivity of histotechnologists and improve times to case readiness. These changes have created a more robust process that is less susceptible to workload and work circumstances variations, as manifest by substantial improvement in the average time of delivery of the last case of the day. We recognize however, that there are still gains to be made in this arena. A favourable impact on worker satisfaction, as well as patient care through improved TAT, is an added benefit to the conventional business case.

Individual histotechnologist productivity data was not available in our baseline state, though future enhancements we plan to incorporate will be able to capture this data.

We have shown productivity improvement similar to those demonstrated by Raab, et al [5] in their multi-year implementation of Lean methods across the histology lab at UPMC, assuming a proportionate allocation of the improvements to each element of the workflow they approached, and that presented in this study. Implementing major changes in process among traditionally manual endeavours may provoke a resistance response among those affected. It is important therefore to have solid baseline data from which to project and demonstrate improvement. This alone creates the case for belief in the new process and thus enlists cooperation rather than apathy, or active resistance. It is this principle which makes it imperative that measurement precede analysis or innovation in conventional Six Sigma and Lean process change.

## Conclusions

The improvements we have documented in our H&E work cell herein described are the beginning of a longer term and broader project to upgrade the performance of our anatomic pathology laboratory. Some quick successes have been realized, but the dramatic overall returns we are seeking will most likely only come from iterative application of the principles of Lean and Six Sigma. We believe that the accrual of small changes with marginal gains will become synergistic as additional changes are implemented, and the culture of change and process improvement is developed. The primary changes we have presented here are limited to one work cell in anatomic pathology, and we intend to continue to revise our processes and integrate that with additional software and technological tools to show continued gains. It is clear that we can continue to reduce the waste in many of our waiting times, and that this can be of benefit to our trainees, pathologists and to our patients. Introduction of an automated staining system alone has produced some small but significant improvements in productivity, and our projections indicate that these will have a reasonable payback period due to significant cost avoidances in additional labor, equipment, space and liability. We believe however, that when combined with other integrated Lean process improvements and technologic tools, such a choice will be even more compelling for those histology labs seeking to enhance the quality, consistency and productivity of their output.

## Competing interests

CY is employed by and holds stock in Roche, which owns Ventana Medical Systems, some of whose products are included in this study. The other authors have no competing interests to disclose.

## Authors' contributions

LH conceived the study, collated and analyzed the data and authored the manuscript. CG participated in process measurements, redesign efforts, and change implementation. CY performed baseline and post-change process measurements, helped redesign process changes and selection of metrics to be used. PE performed data collection and directed day to day implementation of changes. All authors have reviewed the manuscript and accept responsibility for its content.

## Pre-publication history

The pre-publication history for this paper can be accessed here:

http://www.biomedcentral.com/1472-6890/10/2/prepub
